# Impact of Long-Term Chemotherapy on Outcomes in Pancreatic Ductal Adenocarcinoma: A Real-World UK Multi-Centre Study

**DOI:** 10.3390/cancers17111896

**Published:** 2025-06-05

**Authors:** Umair Mahmood, Joanna Lynch, Simran Kaur Sandhu, Zahir Amin, John Bridgewater, Daniel Hochhauser, Kai-Keen Shiu, Paul Miller, Elizabeth C. Smyth, Khurum Khan

**Affiliations:** 1 Department of Gastrointestinal Oncology, University College London Hospitals NHS Foundation Trust, London NW1 2BU, UK; umair.mahmood1@nhs.net (U.M.); john.bridgewater@nhs.net (J.B.); daniel.hochhauser@nhs.net (D.H.); kaikeen.shiu@nhs.net (K.-K.S.); 2 Department of Oncology, Oxford University Hospitals NHS Foundation Trust, Oxford OX3 7LE, UK; paul.miller@ouh.nhs.uk (P.M.); elizabeth.smyth@ouh.nhs.uk (E.C.S.); 3 HCA Healthcare UK, London W1G 6AF, UK; joanna.lynch@hcahealthcare.co.uk (J.L.); simran.kaursandhu@hcahealthcare.co.uk (S.K.S.); 4 Department of Radiology, University College Hospital NHS Foundation Trust (UCLH), London NW1 2BU, UK; zahir.amin@nhs.net; 5 University College London Cancer Institute, London WC1E 6DD, UK

**Keywords:** pancreatic ductal adenocarcinoma, long-term chemotherapy, prolonged survival

## Abstract

**Background:** We reviewed outcomes of short and long-term chemotherapy with or without breaks in pancreatic ductal adenocarcinoma (PDAC) patients. **Methods:** PDAC patients receiving ≥3 chemotherapy cycles between 2019 and 2024 at three institutions were included. Progression-free survival after first-line chemotherapy (PFS1), overall survival (OS) and best overall response (BOR) to chemotherapy were assessed using the Wilcoxon test, Kaplan–Meier test, and univariate and multivariate Cox regression models. **Results:** We screened 237 patients, and 135 patients met the study criteria. Among these patients, 25 had resectable disease, and 110 had unresectable/metastatic disease (13% borderline resectable (BRPC), 20% locally advanced (LAPC), 10% localised developing metastases, 57% de novo metastatic). Ten patients (7%) underwent genetic profiling; *KRAS* aberrations (N = 4), actionable *PLAB2/BRCA2/FGFR2* mutations (N = 3), *ATM/BRIP1* alteration (N = 1). Two patients were managed with PARP inhibitors after receiving multiple lines of chemotherapy. Median PFS1 and OS were concordant with the published literature, but select patient groups achieved prolonged survival outcomes. Among the 36 BRPC/LAPC patients, we observed >1-year PFS1 in 9 (25%) patients and >2-year OS in 3 (8%) patients. Among the 63 de novo metastatic patients, we observed >1-year PFS1 and >2-year OS in 6 (10%) patients. Among patients with localised disease, smoking history was a poor prognostic factor with respect to OS (*p* = 0.03). Improved PFS1 and OS was associated with ≥6 cycles of first-line chemotherapy, its duration of ≥3.66 months, and local treatment after first chemotherapy (*p* < 0.05 for all). Stereotactic body radiotherapy following first-line chemotherapy was delivered in N = 6 (27%) and N = 1 (7%) of patients with LAPC and BRPC, respectively. Chemotherapy interruption duration, but not number, was associated with PFS1 and OS only in the localised cohort (*p* < 0.05). In patients with de novo metastatic disease, prevalence of type 2 diabetes was adversely associated with OS (*p* = 0.03). Improved PFS and OS was associated with ≥6 cycles of first-line chemotherapy, its duration of ≥4.37 months, and BOR to it (only in this cohort) (*p* < 0.05 for all). A favourable OS was associated with >1 line of chemotherapy (*p* = 0.003). **Conclusion:** Despite challenges, extended chemotherapy and multiple treatment lines may improve survival, with localised treatments benefiting select patients.

## Simple Summary

Traditional chemotherapy for pancreatic ductal adenocarcinoma has limited effectiveness and high toxicity, with poor survival outcomes after diagnosis. While best supportive care is often used after initial treatment failure, younger, healthier patients may benefit from extended chemotherapy using alternative regimens. This multi-centre retrospective study examined long-term chemotherapy use in pancreatic ductal adenocarcinoma, finding that patients receiving more treatment lines and longer therapy—potentially due to better tolerance in younger patients—had improved survival. Localised treatment in select patients also contributed to improved survival. Notably, the duration of first-line chemotherapy interruptions, rather than their frequency, was associated with better outcomes in localised cases. Although toxicity remains a concern, managing treatment delays carefully may allow more patients to benefit from extended chemotherapy. Following validation of our results in future studies, our findings may help guide clinicians in selecting patients for long-term treatment and adapting chemotherapy plans to improve survival in this challenging clinical population.

## 1. Introduction

Conventional chemotherapy for pancreatic ductal adenocarcinoma (PDAC) has limited efficacy, significant toxicity, and drug resistance, highlighting the need for new treatment approaches to meet this major unmet clinical need [1,2,3]. PDAC is the third leading cause of cancer death [4], with median overall survival (OS) ranging from 8.5 months with nab-paclitaxel/gemcitabine [5] to 11.1 months with FOLFIRINOX [6]. NICE guidelines recommend first-line FOLFIRINOX for locally advanced or metastatic PDAC with an Eastern Cooperative Oncology Group (ECOG) performance status of 0–1 [7], typically for 12 cycles over 6 months. However, many patients can only tolerate first-line therapy and die within 6–10 months of their diagnosis. Best supportive care is typically considered to maintain quality of life following disease progression after 6 months of chemotherapy. In younger patients with a good performance status, physicians may continue treatment beyond progression by switching to alternative chemotherapeutic regimens to prolong survival. However, there is a paucity of data on outcomes in patients treated with long-term chemotherapy.

We conducted a multi-institutional review of PDAC patients treated with chemotherapy to determine if long-term chemotherapy improves outcomes compared to shorter courses with or without treatment breaks. The study assessed treatment response, progression-free survival (PFS), and OS and sought to identify clinicopathological factors associated with PFS and OS to refine patient selection for chemotherapy. We aimed to identify patients who would benefit most from extended chemotherapy, given the potential for long-term survival benefit in this challenging population.

## 2. Material and Methods

### 2.1. Patient Selection

We screened adult individuals diagnosed with PDAC to identify eligible subjects treated with any type of chemotherapy between 1 January 2019 and 30 June 2024 across three UK healthcare institutions. Patients must have received at least 3 cycles of first-line chemotherapy as standard of care treatment exclusive of participation in an interventional chemotherapy-related clinical trial. We abstracted data from patient medical records including clinician’s notes, radiology, radiation oncology, and operative and pathology reports, as well as clinical reports regarding next-generation sequencing.

### 2.2. Statistical Considerations

Primary outcome measures included progression-free survival following first-line systemic chemotherapy treatment (PFS1) due to the availability of an adequate sample size. PFS1 was defined as the time between the first dose of chemotherapy until radiological/clinical evidence of disease progression, date of last follow-up, or death. We also evaluated OS in our complete cohort. Best overall response (BOR) was evaluated based on the individual physician assessment of chemotherapy response.

Median and interquartile range were used to summarise continuous variables of age, number of cycles, and duration of treatment with chemotherapy, as well as number and duration of chemotherapy interruptions. Frequency tables summarised categorical variables including gender, smoking status, ECOG performance status, stage at initial diagnosis, anatomic site of metastasis, pre-existing co-morbidities, prior surgical treatment, type of chemotherapies received by patients, BOR to each chemotherapy, nature of chemotherapy continuity, common reasons for chemotherapy treatment interruptions, and patterns of systemic treatment change. Outcomes were analysed using Kaplan–Meier actuarial survival methods and Cox proportional-hazards models. Analysis was also performed using univariate and multivariate methods. The Wilcoxon test was used to compare treatment subgroups. Statistical significance was defined as *p* < 0.05 using log-rank tests to identify associations between patient and treatment variables with PFS1 and OS. Patients with missing data were excluded from analysis. All data analysis was performed using R (version 4.2.3) in RStudio (version 2022.12.0.353) and Microsoft Excel (version 16.75) software.

## 3. Results

### 3.1. Clinical and Molecular Profile of PDAC Patient Population

We screened 237 patients and identified 135 (57%) eligible subjects who received chemotherapy for pancreatic ductal adenocarcinoma. The median age at PDAC diagnosis was 65 years, and most patients were female (n = 72, 53%) and were non-smokers (n = 73, 54%) (Table 1). We note a relative higher incidence of patients with an ECOG performance status 0–1 prior to commencing first-line chemotherapy (n = 83, 61%) (Table 1). Most patients presented with de novo metastatic disease at initial diagnosis (n = 63, 47%), primarily in the liver (n = 44, 70%) (Table 1). A limited number of subjects in our cohort were previously managed surgically, mainly individuals presenting with resectable disease (n = 14, 10%) (Table 1). In terms of pre-existing co-morbidities prior to PDAC diagnosis, approximately half the cohort was composed of patients with an abnormal body weight (n = 64, 48%) (Table 1).

There were 10 patients (7%) who underwent genetic profiling on patient request or previous clinical trial screening for exploratory analysis; *KRAS* aberrations (N = 4), actionable *PLAB2/BRCA2/FGFR2* mutations (N = 3), *ATM/BRIP1* alteration (N = 1). Tumour mutational burden was available for a total of six patients; zero mutations per megabase in two patients, three mutations per megabase in one patient, and four mutations per megabase for the remaining three subjects.

We evaluated our patients up to three lines of chemotherapy. First-, second-, and third-line chemotherapy were received by 135, 44, and 9 patients, respectively. The vast majority of our patients received FOLFIRINOX (n = 85, 63%) as their first line of systemic treatment, followed by gemcitabine and nab-paclitaxel in the second-line setting (n = 18, 41%) and fluorouracil and liposomal irinotecan (n = 5, 56%) as the third-line treatment (Supplementary Table S1).

A variable pattern of chemotherapy reception and treatment interruptions was noted across the three systemic treatment lines (Supplementary Table S2A–C). Accumulation of chemotoxicities was the major cause of treatment interruptions in the first (n = 69, 51%) and second treatment line settings (n = 11, 26%) (Supplementary Table S2A,B). Acquiring a COVID-19 infection served as the main cause of third-line chemotherapy interruption (n = 2, 67%) (Supplementary Table S2C). Patterns of chemotherapy change were influenced by the development of progressive disease in our cohort, resulting in a treatment switch to another chemotherapy regimen in the first- (n = 46, 34%) and third-line settings (n = 4, 44%) or systemic treatment discontinuation in the second-line chemotherapy setting (n = 11, 25%) (Supplementary Table S2A–C).

### 3.2. Survival Outcomes of Patients with Unresectable or Metastatic Disease

Our cohort included 25 patients with resectable disease who were excluded from analysis. Among these patients, 10 remained relapse-free after adjuvant chemotherapy, and 9 were alive at 2 years follow-up surveillance. The final analysis included 110 patients with unresectable or metastatic disease (13% borderline resectable, 20% locally advanced, 10% localised disease that developed metastases, and 57% de novo metastatic). As expected, de novo metastatic patients had lower median PFS1 and OS compared to those with localised disease (Table 2).

### 3.3. Treatment Response and Survival Outcomes in the Localised Disease Cohort

Our localised disease cohort included patients with borderline resectable or locally advanced PDAC receiving chemotherapy. A trend towards treatment response or disease stabilisation was noted primarily in the first-line setting (Supplementary Table S3A), with reduced clinical benefit in subsequent lines due to limited patient numbers in these advanced treatment cohorts (Supplementary Table S3B,C).

Multivariate analysis did not demonstrate any association between PFS1 and age (*p* = 0.83), gender (*p* = 0.46), smoking history (*p* = 0.72), ECOG performance status (*p* = 1.00), history of type 2 diabetes (*p* = 0.68), overweight (*p* = 0.37), and obesity (*p* = 0.12) (Table 3). All these patient-specific factors were not associated with OS, with the exception of smoking history (*p* = 0.03) (Table 3).

**Figure 1:**
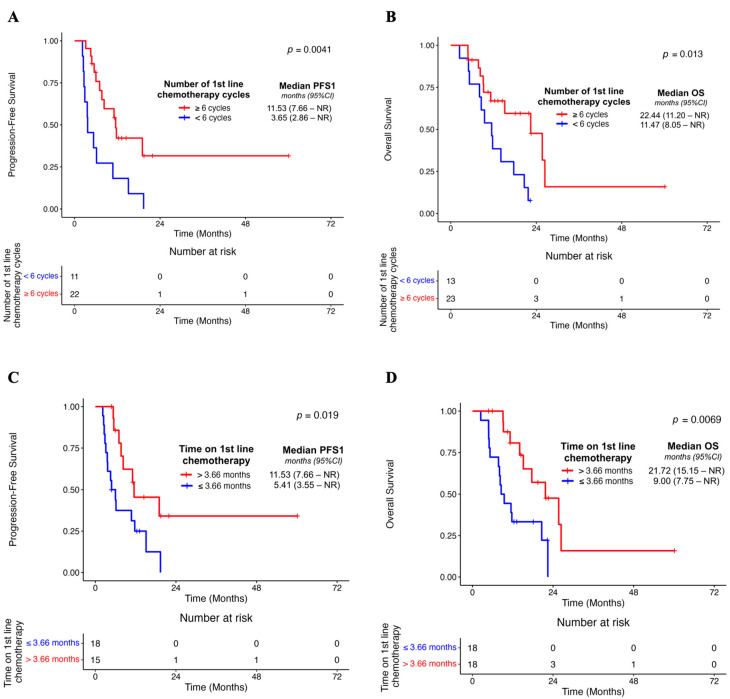
Associations between the number of first-line chemotherapy cycles in the localised disease cohort and PFS1 (**A**) and OS (**B**), respectively. The median value of 6 cycles was used to divide subjects in this disease setting. Associations were also evaluated between the time of first-line chemotherapy in the localised disease cohort and PFS1 (**C**) and OS (**D**), respectively. The median value of 3.66 months was used to divide subjects in this instance.

Patients receiving a greater number of chemotherapy treatment lines were not associated with OS in the localised disease cohort (*p* = 0.44) (Table 3). However, in the first-line setting, a multivariate assessment of treatment-specific factors revealed that an improved PFS1 was associated with ≥6 cycles of first-line chemotherapy (*p* = 0.004), as well as OS (*p* = 0.02) (Figure 1A,B, Table 3). We also observed an association between the median duration of first-line chemotherapy exceeding 3.66 months and PFS1 (*p* = 0.002) as well as OS (*p* = 0.01) (Figure 1C,D, Table 3). During first-line treatment, the median number of chemotherapy interruptions was not associated with PFS1 (*p* = 0.33) or OS (*p* = 0.37) (Table 3). However, we note that patients with a shorter duration of chemotherapy interruption (< 28 days) were associated with poor PFS1 (*p* = 0.02) and OS outcomes in the adjusted models (*p* < 0.001) (Table 3). There was no association between BOR on first-line chemotherapy and PFS1 (*p* = 0.05) or OS (*p* = 0.10) in the adjusted analysis (Table 3). Lastly, we observed an association between local treatment after commencing first-line chemotherapy and PFS1 (*p* < 0.001) as well as OS (*p* < 0.001) (Supplementary Figure S1A,B, Table 3). Given the limited number of patients receiving variable types of localised therapies, caution is warranted while interpreting this hypothesis-generating finding.

### 3.4. Treatment Response and Survival Outcomes in the De Novo Metastatic Disease Cohort

In the de novo metastatic disease cohort, a trend towards decreasing treatment response and disease stabilisation rate was noted while transitioning towards subsequent systemic chemotherapy lines (Supplementary Table S3A–C).

Additionally, our multivariate analysis of patient-specific characteristics revealed no associations between PFS1 and age (*p* = 0.09), gender (*p* = 0.78), smoking history (*p* = 0.72), ECOG performance status (*p* = 0.31), history of type 2 diabetes (*p* = 0.37), overweight (*p* = 0.33), and obesity (*p* = 0.69) (Table 4). Likewise, these factors also did not demonstrate an association with OS, except for a history of type 2 diabetes (*p* = 0.03) (Table 4).

**Figure 2:**
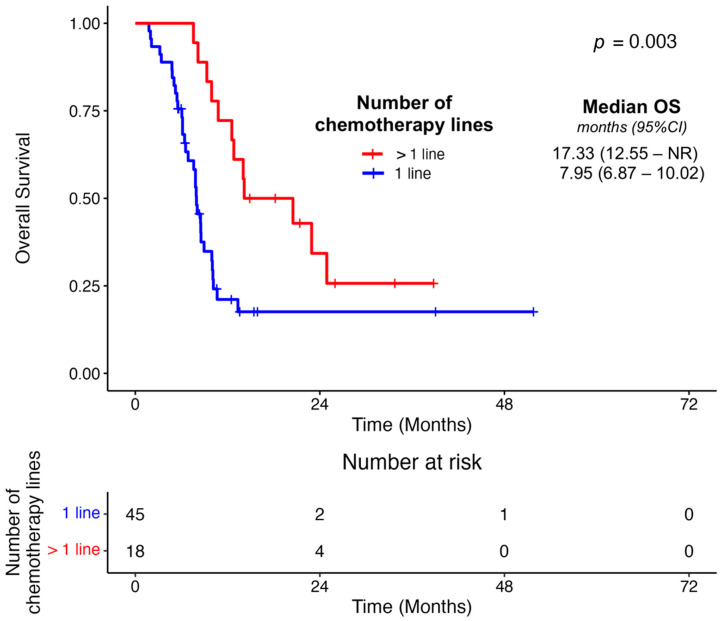
Association between the number of sequential systemic lines of chemotherapy in the de novo metastatic cohort and OS.

However, patients receiving more than a single line of chemotherapy were associated with improved OS outcomes (*p* = 0.003) (Figure 2 and Table 4). Patients receiving a median number of ≥6 cycles of first-line chemotherapy achieved improved PFS1 (*p* < 0.001) and OS (*p* = 0.01) (Supplementary Figure S2A,B and Table 4). Subjects receiving ≥6 cycles of first chemotherapy were younger than patients tolerating fewer cycles (median age 59.84 vs. 72.25 years) (*p* < 0.001) (Supplementary Figure S2C). We also noted that subjects receiving first-line chemotherapy for a period greater than the median duration of 4.37 months experienced prolongation of their PFS1 (*p* < 0.001) and OS (*p* < 0.001) as compared to individuals receiving chemotherapy for ≤4.37 months (Supplementary Figure S3A,B and Table 4). Conversely, PFS1 was not associated with the median number of chemotherapy interruptions (*p* = 0.35) or the duration of such interruptions (*p* = 0.97) in a first-line setting (Table 4). These two factors were also not associated with OS; *p* = 0.46 and *p* = 0.35, respectively (Table 4). Finally, we observed an association between PFS1 and BOR on first-line chemotherapy (*p* = 0.008), as well as OS (*p* = 0.004) (Supplementary Figure S4A,B and Table 4).

### 3.5. Long-Term Chemotherapy Promotes Instances of Exceptional Responses and Prolongation of OS

We observed three subjects who attained an exceptional treatment response and survival outcomes following clinical management of their underlying PDAC with long-term chemotherapeutic regimens.

Patient #135 is a 73-year-old male who completed 6 months of capecitabine and gemcitabine treatment following R1 resection of his pancreatic primary in July 2022. He then completed 12 cycles of FOLFIRINOX for his local disease recurrence in July 2023. This remained stable until January 2024 where the patient was then treated with radiotherapy for his second local recurrence. The subject experienced peritoneal relapse in June 2024 for which the patient received a PARP inhibitor owing to ATM S28822* and BRIP1 W448* mutations, with continued disease stabilisation as of February 2025. Therefore, clinical management using a combination of localised treatment options and multiple lines of systemic therapies (predominantly chemotherapy) resulted in an OS of over 39 months in this patient.

Subject #68 is a 59-year-old male initially diagnosed with localised PDAC who received surgical resection of the primary tumour following completion of 12 cycles of first-line FOLFIRINOX and achieved complete radiographic response (Supplementary Figure S5A,B). The patient subsequently developed disease progression in the liver and was managed with radiotherapy and multiple lines of chemotherapy, resulting in long-term survival exceeding 31 months.

Patient #113 is a 33-year-old female with metastatic liver lesions who achieved a significant treatment response following completion of 13 cycles of modified FOLFIRINOX as first-line chemotherapy (Supplementary Figure S6A,B). She then developed disease progression in a single liver lesion which was managed with radiofrequency ablation and further lines of chemotherapy, resulting in an OS of over 21 months.

In the localised disease cohort, we observed 17 patients whose OS exceeded 12 months, 3 of whom had an OS beyond 24 months. In addition to management with local radiotherapy and surgery after commencing first-line chemotherapy, this improvement in 1-year OS outcomes could have been promoted by the prescription of long-term chemotherapy in a first-line setting composed of a median number of seven cycles and a treatment duration of 5.06 months. Similarly, in the de novo metastatic disease group, 20 subjects had an OS surpassing 12 months, including 6 patients who lived for more than 24 months. This improvement in 1-year OS outcomes can be partially explained by the use of extended first-line chemotherapy, with a median of 12 cycles and a chemotherapy duration of 5.19 months.

## 4. Discussion

Locally advanced or metastatic PDAC represents an important avenue of ongoing clinical investigations to bridge a major unmet clinical need via establishment of treatment standards ensuring optimum survival outcomes. Long-term chemotherapy can improve symptom management, quality of life, disease control, overall survival, and future clinical trial opportunities. We endeavoured to evaluate clinical outcomes of extended chemotherapy in PDAC patients, as data on this population is limited.

Most of our cohort had an ECOG performance status ≤ 1 and received first-line FOLFIRINOX. The PRODIGE-4 trial demonstrated that FOLFIRINOX outperforms gemcitabine in metastatic PDAC, with a median OS increase of 4.3 months (11.1 vs. 6.8 months) [6]. This is partly due FOLFIRINOX patients tolerating more chemotherapy cycles (median 10 vs. 6, *p* < 0.001), but they also experienced more grade 3/4 neutropenia, febrile neutropenia, thrombocytopenia, diarrhoea, and sensory neuropathy. Chemotherapy interruptions did not correspond with survival in our de novo metastatic cohort, suggesting that maintenance chemotherapy, rather than dose density, might be more important and influences survival outcomes. This signifies the importance of active dose interruptions and timely reductions to ensure durable efficacy of extended chemotherapy. Together with PRODIGE-4, our data supports prolonged chemotherapy, with judicious consideration of chemotoxicities, as a prudent first-line approach in metastatic PDAC, preferably in younger patients with ECOG performance status 0–1 as per ESMO guidelines [8].

Neoadjuvant chemotherapy for locally advanced PDAC (LAPC) has gained momentum in recent years [9,10]. Previous real-world evidence demonstrates that most patients with LAPC completing 6 months of neoadjuvant chemotherapy had a median OS not reached in resectable cases and 15.4 months in unresectable cases [11]. Further studies utilising total neo-adjuvant therapy for borderline resectable or locally advanced PDAC have demonstrated that treatment with ≥6 cycles of chemotherapy is an independently associated factor with survival [12]. Given the increasing interest in neoadjuvant treatments, it would be important to further explore the utility of extended chemotherapy within this disease setting using prospectively designed studies.

The low incidence of actionable genetic alterations in PDAC (0.5–7%) [13] often obviates the need for comprehensive genomic profiling in standard clinical practice. Limited profiling in our cohort was driven by patient request or previous clinical trial screening. However, KRAS alterations are noted in 80–93% of PDAC cases in previous studies [14,15], prompting exploration of RAS inhibitors in ongoing clinical trials. Successful developments originating from these initiatives would confer a paradigm shift in management of PDAC patients owing to a synergistic effect by combining extended chemotherapy treatment plans with targeted agents to improve patient outcomes.

Our study is limited by heterogeneity in metastatic anatomic sites and variability in treatment types, doses, and schedules. Subgroup sample sizes were limited precluding further stratification in our analysis accounting for additional variables, and multiple testing adjustments were not performed. Genomic profiling was performed on a limited number of samples, and the results were exploratory in nature. We intended to limit selection of patients receiving ≥3 cycles of first-line chemotherapy to allow for adequate follow-up time to perform PFS analysis and treatment response assessment. However, this patient selection strategy might have also influenced our results by overestimating our survival outcomes. We observed a relatively lower median age of patients who commenced first-line FOLFIRINOX/modified FOLFIRINOX (N = 79) vs. other chemotherapy regimens (N = 31) (64 years vs. 75 years). Hence, selection of younger patients who were able to better tolerate FOLFIRINOX/modified FOLFIRINOX could have impacted our study findings. It was also challenging to account for chemotherapy dose adjustments as well as reductions between full-dose and milder regimens as these can tend to vary between institutions. Most patients received the outdated FOLFIRINOX regimen, associated with greater side effects, poor compliance, and higher treatment discontinuation, leading to suboptimal outcomes [16,17,18]. With the recent shift towards modified FOLFIRINOX, we would anticipate better survival outcomes than observed in our study due to improved chemotolerance, a hypothesis that warrants further investigation in future studies. Our study did not include detailed characterisation of patients receiving localised therapies. Such characterisation and further evaluation of localised therapies impacting outcomes in the context of PDAC management is an important concept which should be explored in future studies specifically designed for this purpose.

## 5. Conclusions

Our data supports consideration of extended chemotherapy for both localised and de novo metastatic PDAC patients. Promising outcomes were seen in patients receiving more chemotherapy lines and longer treatment, potentially due to better tolerance in younger patients. Achieving systemic response contributes to favourable PFS and OS outcomes. While limited genomic profiling was performed, localised treatments in carefully selected patients may improve survival. Chemotherapy-related toxicity is a concern, but careful treatment delays may allow PDAC patients to remain on treatment and derive the clinical benefits associated with long-term chemotherapy. Despite limitations of a retrospective analysis, our findings could help guide clinicians to aid patient selection for long-term chemotherapy or encourage modification of chemotherapy plans to optimise clinical outcomes in this challenging patient population. Future investigations could further explore our findings in larger prospectively designed clinical trials to further validate our results.

## Figures and Tables

**Table 1:** Baseline characteristics of the complete cohort (N = 135).

	Total Number of Subjects	
	N	%
**Age at commencing first-line chemotherapy following PDAC diagnosis (years)**		
Median	65	
Range	33–85	
Interquartile range	60–73	
**Gender**		
Male	63	47
Female	72	53
**Smoking status**		
Non-smoker	73	54
Current smoker	13	10
Former smoker	38	28
Unknown	11	8
**ECOG performance status**		
ECOG 0	39	29
ECOG 0–1	2	1
ECOG 1	42	31
ECOG 1–2	6	4
ECOG 2	7	5
Unknown	39	29
**Stage at initial diagnosis**		
Resectable PDAC	14	10
Borderline resectable PDAC	14	10
Locally advanced PDAC	22	16
Localised disease followed by distant metastases *	22	16
De novo metastatic disease	63	47
**Anatomic site of disease for de novo metastatic patients**		
Lung	18	29
Liver	44	70
Brain	1	2
Bone	3	5
Lymph node	12	19
Peritoneum	6	10
Other **	14	22
Prior surgical treatment ***		
Resectable PDAC	14	10
Borderline resectable PDAC	2	1
Locally advanced PDAC	1	1
Localised disease followed by distant metastases	11	8
De novo metastatic disease	2	1
**Pre-existing co-morbidities prior to PDAC diagnosis**		
Type 2 diabetes mellitus	23	17
Chronic pancreatitis	3	2
Abnormal body weight		
*Overweight*	32	24
*Obese*	32	24
*None*	47	35
*Unknown*	24	18

Abbreviations: ECOG, Eastern Cooperative Oncology Group; PDAC, pancreatic ductal adenocarcinoma. * Refers to patients receiving chemotherapy both at the time of initial localised disease and subsequently after metastatic progression. ** Omental metastasis (N = 3), adrenal gland (N = 2), spleen (N = 2), mesentery of the small bowel and omentum (N = 1), appendical mass (N = 1), gallbladder (N = 1), ovary (N = 1), lesion located inferior to the third part of the duodenum (N = 1), stomach (N = 1), colon (N = 1). *** None of the evaluated patients received radiotherapy prior to commencing first-line chemotherapy.

**Table 2:** Survival outcomes in major diagnostically staged unresectable cohorts (N = 110).

	Median PFS1 *months* (95% CI)	Median OS *months* (95% CI)
Localised disease * (N = 36)	8.28 (5.98–19.02)	15.15 (11.20–NR)
**Localised disease followed by distant metastases (N = 11)**	8.15 (2.56–NR)	18.27 (17.38–NR)
**De novo metastatic disease (N = 63)**	6.41 (5.98–8.18)	9.30 (8.05–12.81)

Abbreviations: CI, confidence interval; NR, not reached; OS, overall survival; PFS1, progression-free survival while on first-line chemotherapy. * Refers to patients diagnosed with borderline resectable and locally advanced pancreatic ductal adenocarcinoma.

**Table 3:** Associations with PFS1 and OS for patients with localised disease (i.e., borderline resectable and locally advanced disease settings).

	PFS1				OS			
	Univariate HR (95% CI)	*p*-Value	Multivariate HR (95% CI) *	*p*-Value	Univariate HR (95% CI)	*p*-Value	Multivariate HR (95% CI) *	*p*-Value
**Age at commencing first chemotherapy** (<75 years vs. ≥75 years)	0.65 (0.21–1.97)	0.45	0.88 (0.28–2.79)	0.83	0.39 (0.14–1.09)	0.07	0.49 (0.16–1.45)	0.20
**Gender** (male vs. Female)	0.69 (0.26–1.82)	0.46	0.68 (0.25–1.88)	0.46	0.96 (0.38–2.41)	0.93	0.94 (0.35–2.53)	0.90
**Smoking status** (current or former smoker vs. never smoker)	1.30 (0.56–3.00)	0.54	1.19 (0.46–3.07)	0.72	2.52 (1.00–6.34)	0.05	3.46 (1.10–10.86)	0.03
**ECOG performance status at commencing first chemotherapy** (ECOG 0 vs. ECOG 1 or higher)	0.00 (0.00–Inf)	1.00	0.00 (0.00–Inf)	1.00	0.00 (0.00–Inf)	1.00	0.00 (0.00–Inf)	1.00
**History of type 2 diabetes** (prevalent vs. non-prevalent)	0.84 (0.28–2.50)	0.76	1.28 (0.39–4.17)	0.68	1.02 (0.30–3.50)	0.97	1.81 (0.47–6.98)	0.39
**History of abnormal body weight**								
None	-	-	-	-	-	-	-	-
Overweight	1.34 (0.42–4.25)	0.62	1.89 (0.47–7.62)	0.37	1.53 (0.48–4.86)	0.47	1.72 (0.52–5.65)	0.37
Obese	2.06 (0.65–6.49)	0.22	2.98 (0.75–11.84)	0.12	2.36 (0.74–7.51)	0.15	2.52 (0.75–8.44)	0.14
**Number of chemotherapy lines** (>1 vs. 1)	N/A	N/A	N/A	N/A	0.87 (0.29–2.59)	0.80	0.63 (0.19–2.04)	0.44
**Number of first-line chemotherapy cycles** (≥6 vs. <6 cycles)	0.32 (0.14–0.72)	0.006	0.23 (0.09–0.63)	0.004	0.34 (0.14–0.83)	0.02	0.22 (0.06–0.77)	0.02
**Duration of first-line chemotherapy** (≥3.66 months vs. <3.66 months) **	0.37 (0.15–0.88)	0.02	0.21 (0.08–0.57)	0.002	0.30 (0.12–0.75)	0.01	0.30 (0.11–0.78)	0.01
**Number of first-line chemotherapy interruptions** (≤2 vs. >2 treatment interruptions)	1.00 (0.41–2.42)	0.99	1.72 (0.58–5.16)	0.33	1.55 (0.56–4.28	0.39	1.67 (0.54–5.11)	0.37
**Duration of first-line chemotherapy interruptions** (≤28 vs. >28 interrupted days)	2.22 (0.90–5.47)	0.08	3.48 (1.20–10.09)	0.02	3.93 (1.26–12.27)	0.02	13.48 (2.90–62.60)	<0.001
**Best overall response on first-line chemotherapy** (responders vs. non-responders)	0.36 (0.14–0.90)	0.03	0.39 (0.15–1.02)	0.05	0.30 (0.11–0.84)	0.02	0.41 (0.14–1.19)	0.10
**Local treatment after commencing first-line chemotherapy** (recipients vs. non-recipients)	0.10 (0.03–0.34)	<0.001	0.09 (0.03–0.32)	<0.001	0.19 (0.07–0.51)	<0.001	0.19 (0.07–0.50)	<0.001

* Adjusted analysis by age at commencing first chemotherapy, gender, local stage at diagnosis, and radiotherapy and/or surgical treatment after commencing first-line chemotherapy. ** Refers to chemotherapy duration while excluding total treatment interruptions. Abbreviations: CI, confidence interval; ECOG, Eastern Cooperative Oncology Group; HR, hazard ratio; N/A, not applicable; OS, overall survival; PFS1, progression-free survival while on first-line chemotherapy.

**Table 4:** Associations with PFS1 and OS in the de novo metastatic disease cohort.

	PFS1				OS			
	Univariate HR (95% CI)	*p*-Value	Multivariate HR (95% CI) *	*p*-Value	Univariate HR (95% CI)	*p*-Value	Multivariate HR (95% CI) *	*p*-Value
**Age at commencing first chemotherapy** (<75 years vs. ≥75 years)	0.64 (0.33–1.23)	0.18	0.54 (0.26–1.11)	0.09	0.52 (0.27–0.99)	0.05	0.55 (0.27–1.15)	0.11
**Gender** (male vs. female)	1.24 (0.71–2.16)	0.44	0.91 (0.48–1.74)	0.78	1.22 (0.68–2.21)	0.50	1.02 (0.51–2.02)	0.96
**Smoking status** (current or former smoker vs. never smoker)	1.51 (0.79–2.89)	0.21	1.14 (0.56–2.32)	0.72	1.74 (0.89–3.41)	0.10	1.14 (0.56–2.36)	0.71
**ECOG performance status at commencing first chemotherapy** (ECOG 0 vs. ECOG 1 or higher)	1.00 (0.51–1.96)	1.00	1.46 (0.71–3.02)	0.31	0.87 (0.44–1.74)	0.70	0.88 (0.41–1.87)	0.74
**History of type 2 diabetes** (prevalent vs. non-prevalent)	0.84 (0.37–1.90)	0.67	1.54 (0.60–3.95)	0.37	1.34 (0.59–3.03)	0.49	2.94 (1.13–7.63)	0.03
**History of abnormal body weight**								
None	-	-	-	-	-	-	-	-
Overweight	0.65 (0.28–1.51)	0.31	0.63 (0.25–1.60)	0.33	0.78 (0.34–1.81)	0.56	0.56 (0.23–1.36)	0.20
Obese	0.95 (0.48–1.87)	0.88	0.85 (0.39–1.86)	0.69	1.05 (0.52–2.13)	0.90	0.74 (0.34–1.61)	0.45
**Number of chemotherapy lines** (>1 vs. 1)	N/A	N/A	N/A	N/A	0.37 (0.18–0.73)	0.004	0.31 (0.14–0.67)	0.003
**Number of first-line chemotherapy cycles** (≥6 vs. <6 cycles)	0.24 (0.13–0.45)	<0.001	0.26 (0.12–0.56)	<0.001	0.28 (0.15–0.51)	<0.001	0.40 (0.20–0.80)	0.01
**Duration of first-line chemotherapy** (≥4.37 months vs. <4.37 months) **	0.25 (0.14–0.45)	<0.001	0.11 (0.05–0.26)	<0.001	0.28 (0.15–0.52)	<0.001	0.23 (0.12–0.45)	<0.001
**Number of first-line chemotherapy interruptions** (≤2 vs. >2 treatment interruptions)	1.30 (0.69–2.44)	0.42	1.38 (0.70–2.74)	0.35	1.39 (0.72–2.69)	0.32	1.30 (0.65–2.58)	0.46
**Duration of first-line chemotherapy interruptions** (≤34 vs. >34 interrupted days)	0.99 (0.52–1.86)	0.96	0.99 (0.51–1.93)	0.97	1.14 (0.59–2.21)	0.69	1.39 (0.70–2.75)	0.35
**Best overall response on first-line chemotherapy** (responders vs. non-responders)	0.32 (0.17–0.59)	<0.001	0.40 (0.21–0.79)	0.008	0.27 (0.14–0.52)	<0.001	0.35 (0.17–0.71)	0.004

*Adjusted analysis by age at commencing first chemotherapy, gender, and local treatment after commencing first-line chemotherapy. ** Refers to chemotherapy duration while excluding total treatment interruptions. Abbreviations: CI, confidence interval; ECOG, Eastern Cooperative Oncology Group; HR, hazard ratio; N/A, not applicable; OS, overall survival; PFS1, progression-free survival while on first-line chemotherapy.

## Data Availability

The original contributions presented in this study are included in the article/Supplementary Materials; further inquiries can be directed to the corresponding author.

## Supplementary Materials

The following supporting information can be downloaded at: https://www.mdpi.com/article/10.3390/cancers17111896/s1, Supplementary Figure S1: Associations between local treatment after commencing 1st line chemotherapy in the localised disease cohort and PFS1 (A) and OS (B), respectively. Supplementary Figure S2: Associations between the number of 1st line chemotherapy cycles in the de novo metastatic cohort and PFS1 (A) and OS (B), respectively. The median value of 6 cycles was used to divide subjects in this disease setting. A boxplot of patient’s age at the time of initiating 1st line chemotherapy in the de novo metastatic cohort with respect to the number of 1st line chemotherapy treatment cycles (C). Supplementary Figure S3: Associations between time on 1st line chemotherapy among de novo metastatic patients and PFS1 (C) and OS (D), respectively. The median value of 4.37 months was used to divide subjects in this instance. Supplementary Figure S4: Associations between best overall response (BOR) on 1st line chemotherapy in the de novo metastatic cohort and PFS1 (A) and OS (B), respectively. Responders achieved partial or complete response as their BOR, while non-responders had stable or progressive disease as their BOR on 1st line chemotherapy. Supplementary Figure S5: Pre-treatment baseline scan for subject # 68 from February 2022 demonstrated a pancreatic primary with largest axial measurement of 25.1 mm (A). Patient achieved complete radiographic response in December 2022 after completing 12 cycles of FOLFIRINOX (B). Supplementary Figure S6: Pre-treatment baseline scan of Subject # 113 from December 2022 demonstrates liver metastases with largest axial measurement of 15.36 mm (A). Patient achieved partial radiographic response in March 2023 while on 1st line modified FOLFIRINOX, which demonstrated the greatest regression in liver tumour volume in July 2023 limited to 2.94 mm (B). Supplementary Table S1: Systemic treatment types stratified by treatment lines among evaluated patients (N = 135). Supplementary Table S2: (A) Systemic treatment details for patients on 1st line chemotherapy (N = 135). (B) Systemic treatment details for patients on 2nd line chemotherapy (N = 44). (C) Systemic treatment details for patient on 3rd line chemotherapy (N = 9). Supplementary Table S3: (A) Best overall response amongst unresectable patients following 1st line treatment with chemotherapy stratified by stage at initial diagnosis (N = 110). (B) Best overall response amongst unresectable patients following 2nd line treatment with chemotherapy stratified by stage at initial diagnosis (N = 33). (C) Best overall response amongst unresectable patient following 3rd line treatment with chemotherapy stratified by stage at initial diagnosis (N = 7).
