# Supplementary material for: Impact of Long-Term Chemotherapy on Outcomes in Pancreatic Ductal Adenocarcinoma: A Real-World UK Multi-Centre Study
Source: Cancers (Basel). 2025 Jun 5;17(11):1896. doi: 10.3390/cancers17111896 (PMC12153574; doi:10.3390/cancers17111896)
Supplement: Supplementary file 1 [file cancers-17-01896-s001.zip › Supplementary Figures.pdf]

A

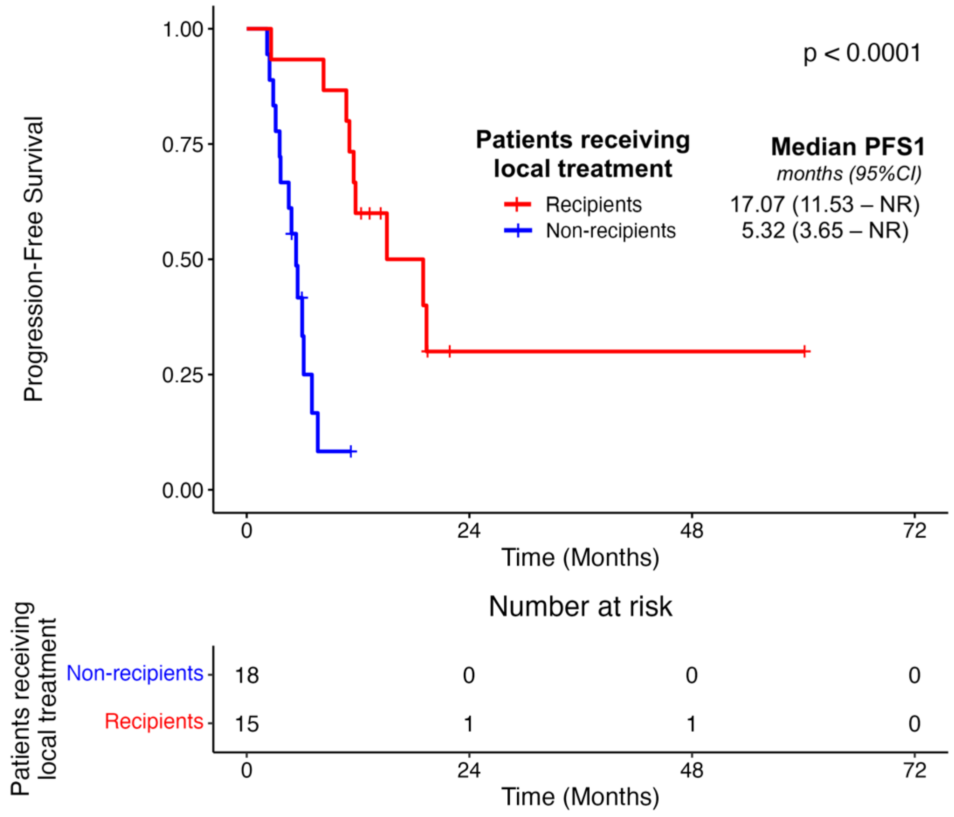

B

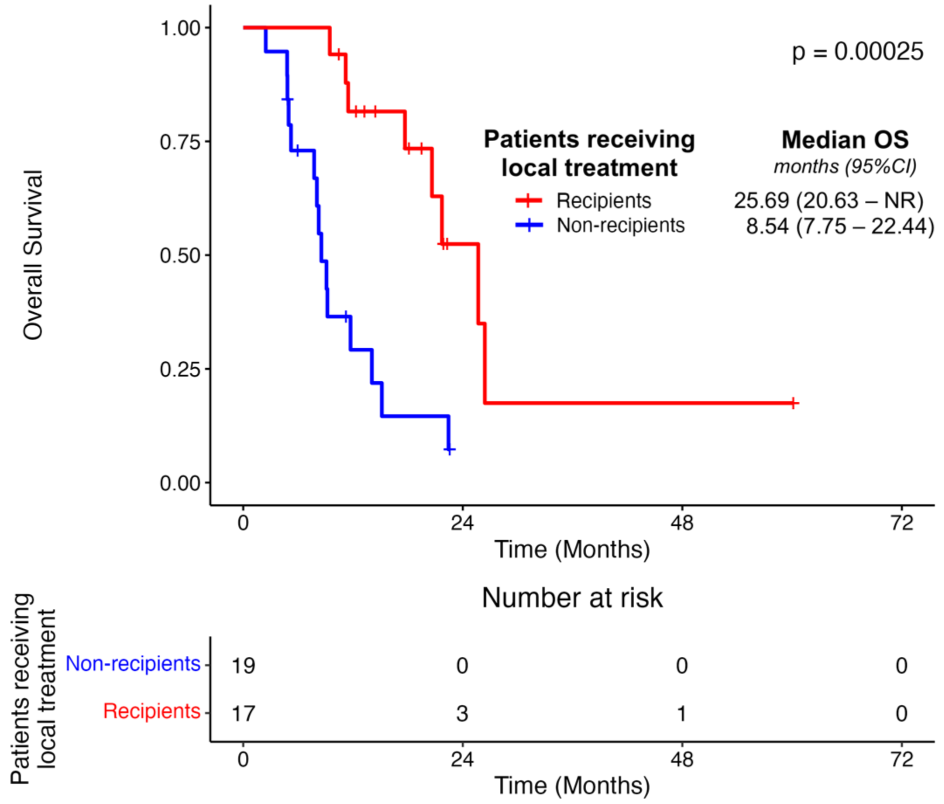

Supplementary Figure S1. Associations between local treatment after commencing 1st line chemotherapy in the localised disease cohort and PFS1 (A) and OS (B), respectively.

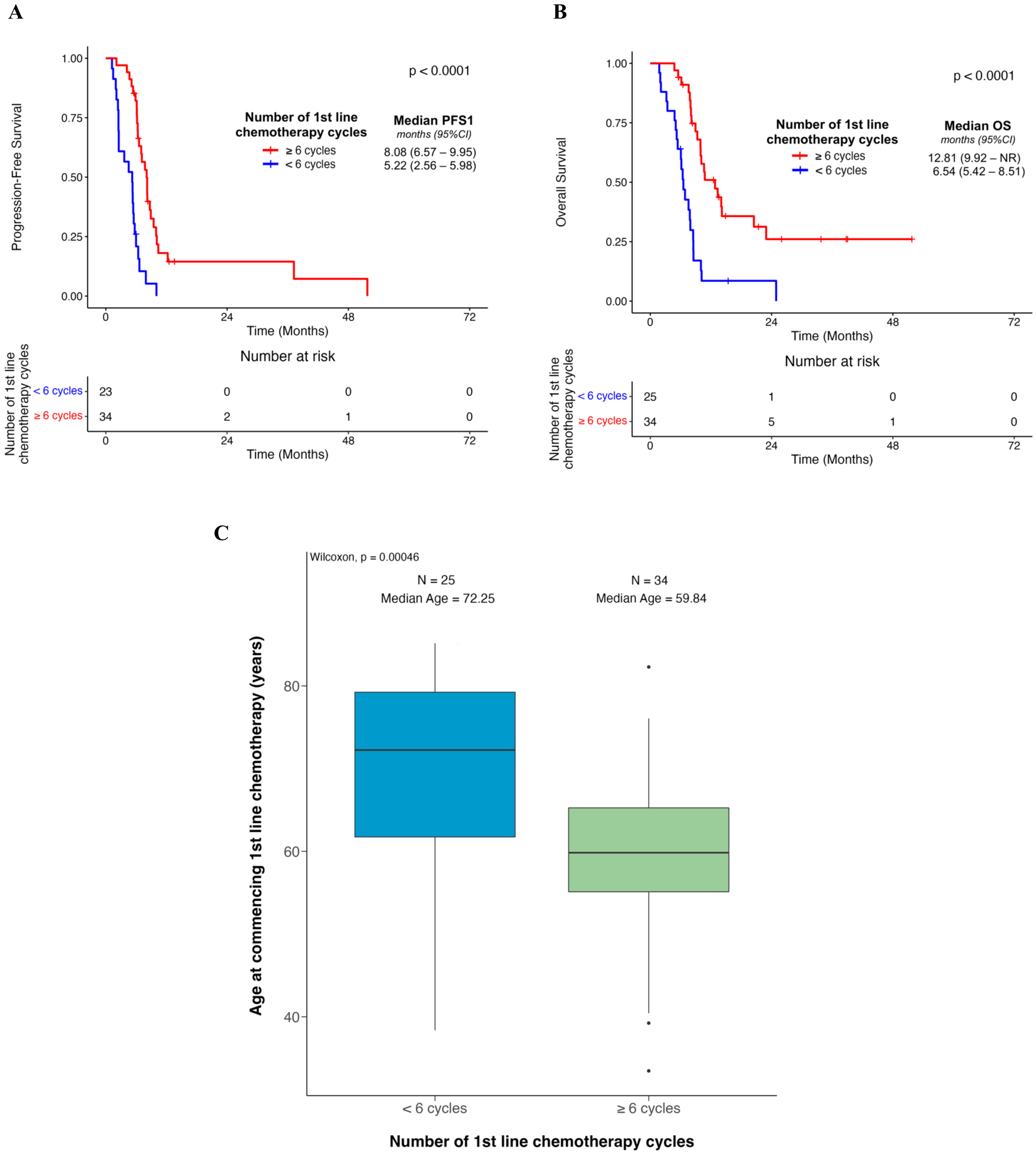

Supplementary Figure S2. Associations between the number of 1st line chemotherapy cycles in the de novo metastatic cohort and PFS1 (A) and OS (B), respectively. The median value of 6 cycles was used to divide subjects in this disease setting. A boxplot of patient's age at the time of initiating 1st line

chemotherapy in the de novo metastatic cohort with respect to the number of 1st line chemotherapy treatment cycles (C).

A

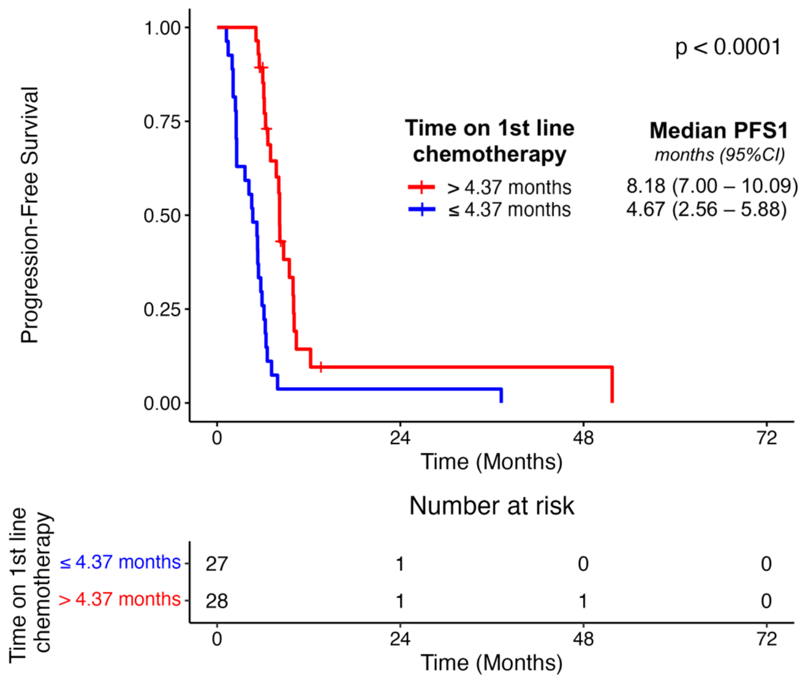

B

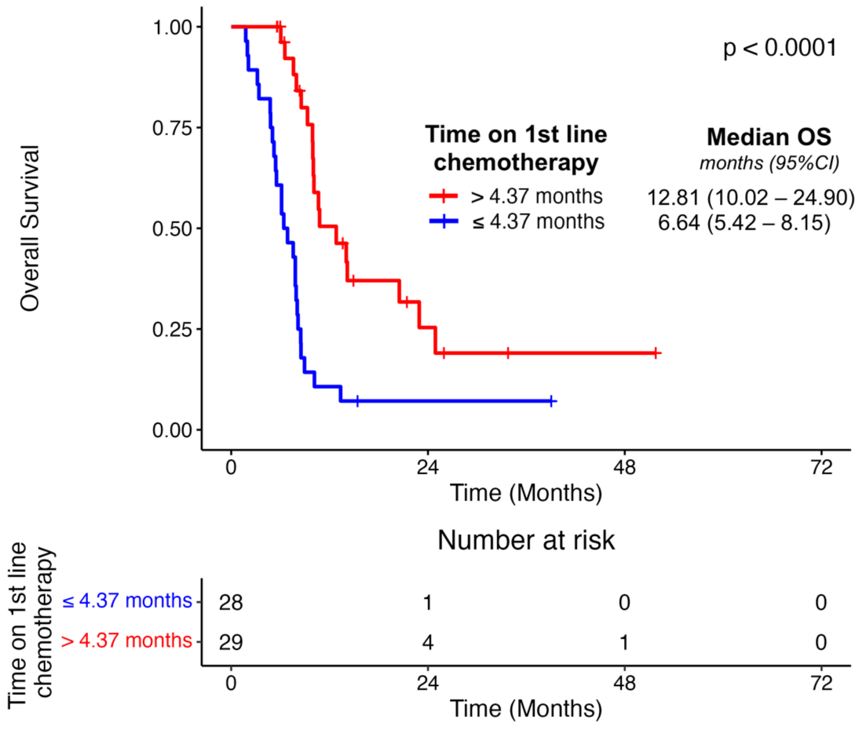

Supplementary Figure S3. Associations between time on 1st line chemotherapy among de novo metastatic patients and PFS1 (C) and OS (D), respectively. The median value of 4.37 months was used to divide subjects in this instance.

A

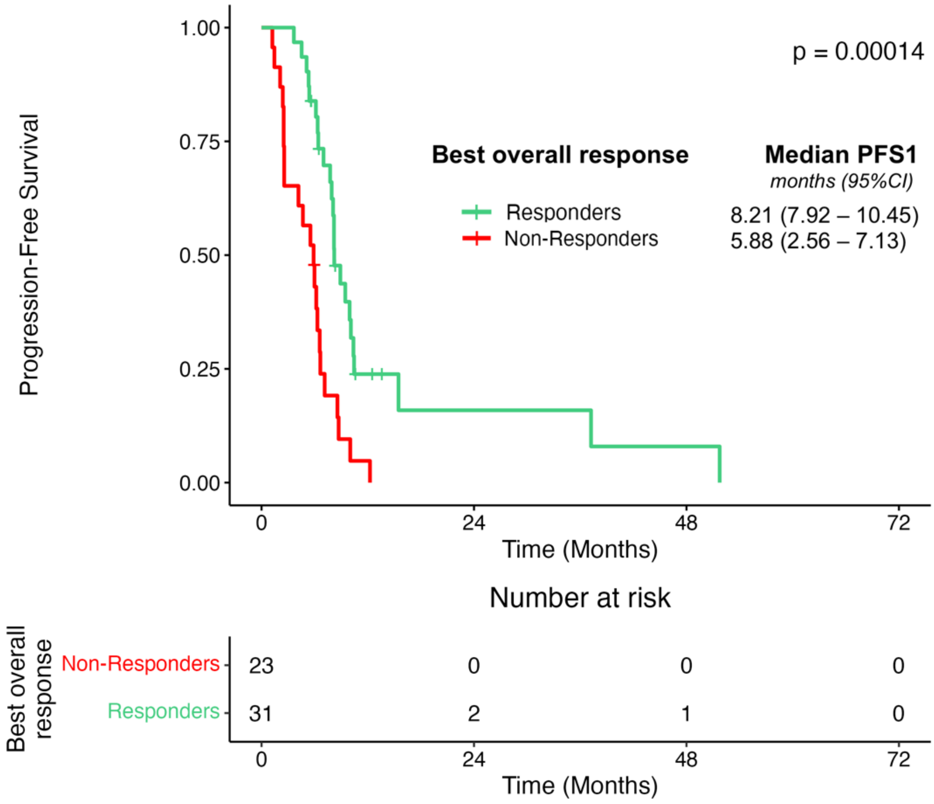

B

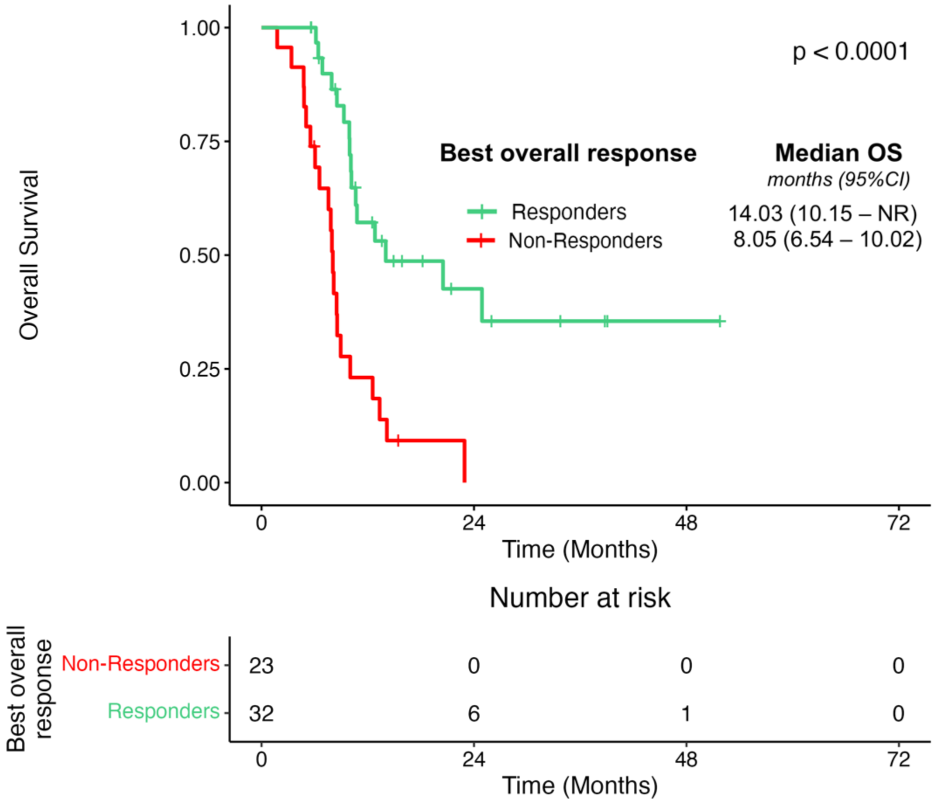

Supplementary Figure S4: Associations between best overall response (BOR) on 1st line chemotherapy in the de novo metastatic cohort and PFS1 (A) and OS (B), respectively. Responders achieved partial or complete response as their BOR, while non-responders had stable or progressive disease as their BOR on 1st line chemotherapy.

A

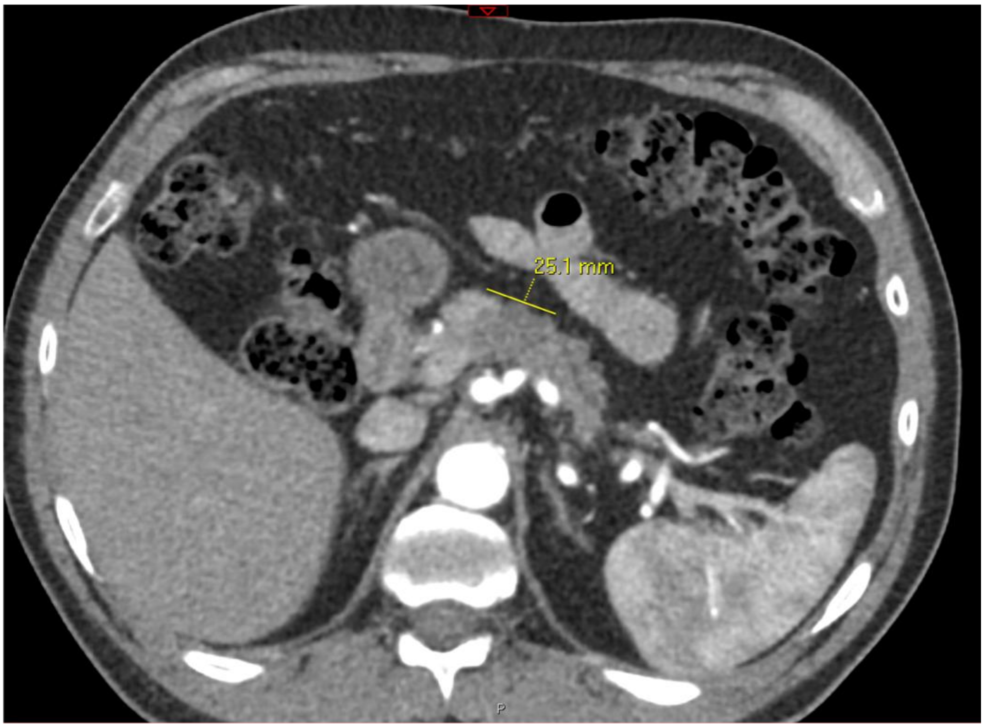

B

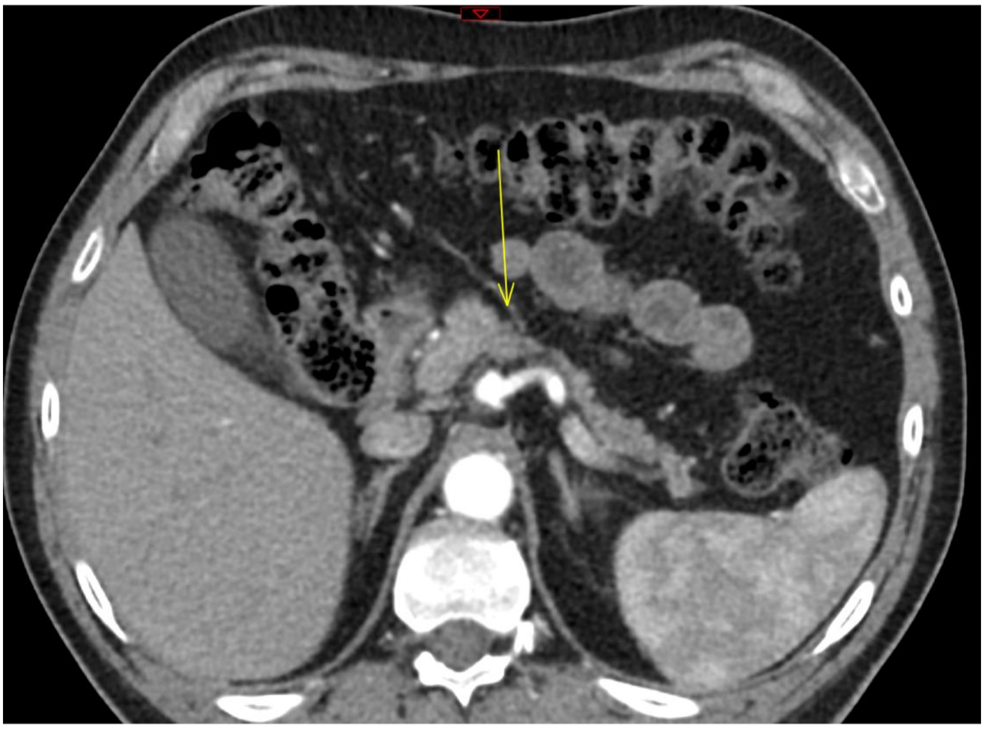

Supplementary Figure S5: Pre-treatment baseline scan for subject # 68 from February 2022 demonstrated a pancreatic primary with largest axial measurement of 25.1 mm (A). Patient achieved complete radiographic response in December 2022 after completing 12 cycles of FOLFIRINOX (B).

A

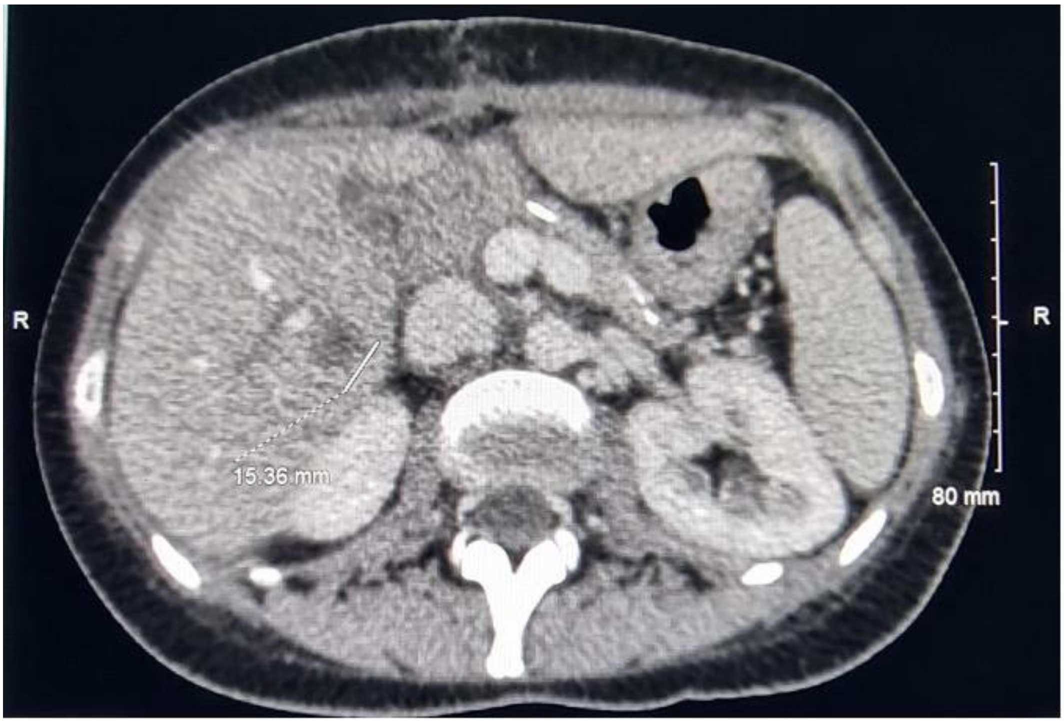

B

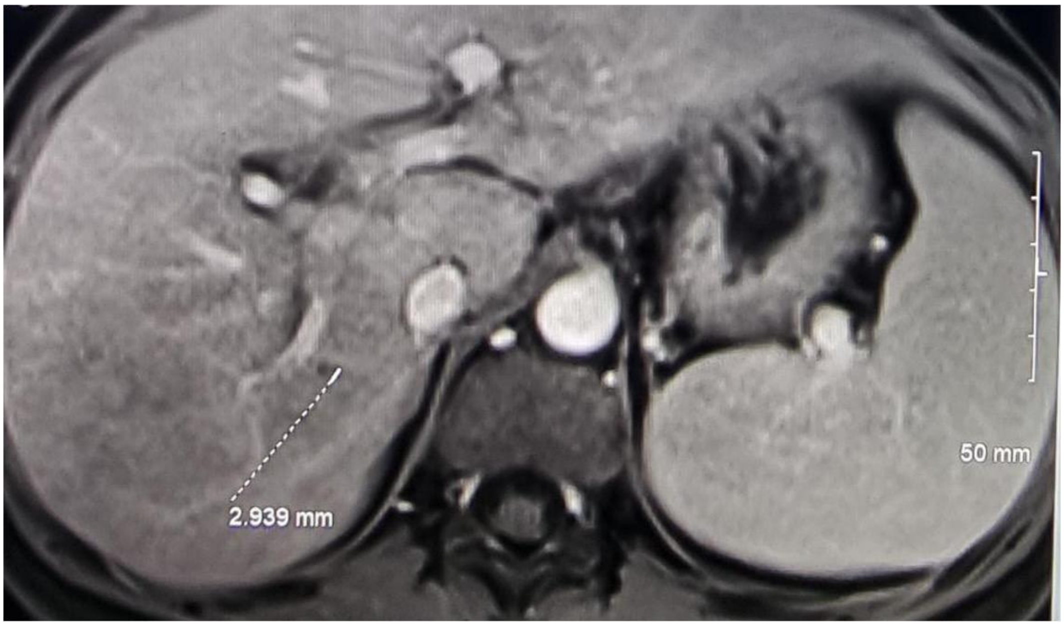

Supplementary Figure S6. Pre-treatment baseline scan of Subject # 113 from December 2022 demonstrates liver metastases with largest axial measurement of 15.36 mm (A). Patient achieved partial radiographic response in March 2023 while on 1st line modified FOLFIRINOX, which demonstrated the greatest regression in liver tumour volume in July 2023 limited to 2.94 mm (B).
