# Supplementary material for: Impact of Long-Term Chemotherapy on Outcomes in Pancreatic Ductal Adenocarcinoma: A Real-World UK Multi-Centre Study
Source: Cancers (Basel). 2025 Jun 5;17(11):1896. doi: 10.3390/cancers17111896 (PMC12153574; doi:10.3390/cancers17111896)
Supplement: Supplementary file 1 [file cancers-17-01896-s001.zip › Supplementary Tables.pdf]

Supplementary Tables

Supplementary Table S1: Systemic treatment types stratified by treatment lines among evaluated patients (N = 135).

	**Total number of subjects**	
	**N**	**%**
**1st line chemotherapy**	**135**	**100**
FOLFIRINOX	85	63
Gemcitabine and nab-paclitaxel	16	12
Gemcitabine	11	8
Modified FOLFIRINOX	9	7
Gemcitabine and capecitabine	8	6
Gemcitabine, capecitabine and nab-paclitaxel	3	2
Acelarin	2	2
FOLFOX	1	1
**2nd line chemotherapy**	**44**	**100**
Gemcitabine and nab-paclitaxel	18	41
Gemcitabine	15	34
FOLFIRINOX	7	16
Gemcitabine and capecitabine	2	5
Capecitabine	2	5
**3rd line chemotherapy**	**9**	**100**
Fluorouracil and liposomal irinotecan	5	56
Gemcitabine and capecitabine	3	33
Gemcitabine	1	11

Abbreviations: FOLFIRINOX, Folinic acid, Fluorouracil, Irinotecan, Oxaliplatin; FOLFOX, Folinic acid, Fluorouracil, Oxaliplatin.

Supplementary Table S2A: Systemic treatment details for patients on 1st line chemotherapy (N = 135).

	Total number of subjects	
	**N**	**%**
**Number of chemotherapy cycles completed**		
Median	6	
Range	3 – 30	
**Duration of total chemotherapy received (months)***		
Median	4.50	
Range	0.82 – 16.59	
**Nature of chemotherapy continuity**	**135**	**100**
Continuous treatment	18	13
Non-continuous treatment	107	80
Unknown	10	8
**Number of chemotherapy interruptions**		
Median	2	
Range	1 – 11	
**Total duration of chemotherapy interruptions (days)**		
Median	27	
Range	3 – 151	
**Common reasons for treatment interruptions**		
Chemotherapy related toxicity	69	51
Non-chemotherapy related hospital admission	21	16
Patient choice (opted for treatment break, vacation, forgot to attend scheduled appointments)	21	16
Physician discretion	13	10
Covid, chest, urinary tract and other infections	10	7
ERCP to treat blocked bile duct stent	6	4
Deranged laboratory results (electrolytes, bilirubin, alanine transaminase and hemoglobin levels)	5	4
Miscellaneous**	17	13
Unknown causes	24	18

**Patterns of change in current chemotherapy or treatment continuation**	**135**	**100**
Discontinued all systemic therapy due to disease progression	22	16
Discontinued all systemic therapy due to declining performance status	8	6
Discontinued all systemic therapy due to chemotoxicity	9	7
Discontinued all systemic therapy due to other reasons***	30	22
Switched to 2nd line chemotherapy due to disease progression	46	34
Switched to 2nd line chemotherapy due to chemotoxicity	2	1
Patient passed away before resuming interrupted treatment due to chemotoxicity or being able to commence 2nd line treatment after developing progressing disease	5	4
Continued current chemotherapy via treatment re-challenge	6	4
Ongoing current chemotherapy as of last follow-up visit	7	5

Abbreviations: ERCP, Endoscopic Retrograde Cholangiopancreatography.
* Refers to actual chemotherapy duration while excluding total treatment interruptions.
** Miscellaneous: Logistical challenges (N = 8), delays in scheduling treatment appointments due to New Year holidays (N = 5), patient too ill to travel to attend chemotherapy appointment (N = 2), broken red skin in hands due to unknown etiology (N = 1), treatment for deep vein thrombosis (N = 1).
*** Other reasons for systemic treatment discontinuation: Disease progression and declining performance status (N = 6), disease progression and chemotoxicity (N = 1), patient opted to pursue palliative care (N = 2), declining performance status and patient choice to pursue hospice care (N = 2), patient opted for surgery and radiotherapy to treat remaining limited/local disease (N = 2), patient completed all adjuvant chemotherapy after surgical resection of pancreatic tumour (N = 8), patient is on surveillance after completing all adjuvant chemotherapy (N = 5), patient is on surveillance after completing 12 cycles of FOLFIRINOX chemotherapy (N = 3), patient opted to pursue high intensity frequency ultrasound treatment (N = 1).

Supplementary Table S2B: Systemic treatment details for patients on 2nd line chemotherapy (N = 44).

	Total number of subjects	
	**N**	**%**
**Number of chemotherapy cycles completed**		
Median	3	
Range	0 – 12	
**Duration of total chemotherapy received (months)***		
Median	2.05	
Range	0.00 – 11.27	
**Nature of chemotherapy continuity**	**44**	**100**
Continuous treatment	7	16
Non-continuous treatment	29	66
Unknown	8	18
**Number of chemotherapy interruptions**		
Median	2	
Range	1 – 9	
**Total duration of chemotherapy interruptions (days)**		
Median	21	
Range	7 – 313	
**Common reasons for treatment interruptions**		
Chemotherapy related toxicity	11	26
Non-chemotherapy related hospital admission	6	14
Patient choice (opted for treatment break, vacation)	9	21
Miscellaneous**	9	21
Unknown causes	8	19
**Patterns of change in current chemotherapy or treatment continuation**	**44**	**100**
Discontinued all systemic therapy due to disease progression	11	25
Discontinued all systemic therapy due to declining performance status	3	7
Discontinued all systemic therapy due to chemotoxicity	2	5
Discontinued all systemic therapy due to other reasons***	7	16

Switched to 3rd line chemotherapy due to disease progression	6	14
Switched to 3rd line chemotherapy due to chemotoxicity	1	2
Opted for radiotherapy to treat remaining local recurrence	1	2
Patient passed away during treatment	3	7
Continued chemotherapy via 1st or 2nd line treatment re-challenge	4	9
Ongoing current chemotherapy as of last follow-up visit	5	11
Ongoing treatment management discussion after progressive disease as of last follow-up visit	1	2

Abbreviations: ERCP, Endoscopic Retrograde Cholangiopancreatography.
* Refers to actual chemotherapy duration while excluding total treatment interruptions.
** Miscellaneous: Covid and other infections (N = 4), deranged laboratory results (bilirubin, alanine transaminase levels) (N = 2), fever (N = 1), ERCP to treat blocked bile duct stent (N = 1), physician discretion (N = 1).
*** Other reasons for systemic treatment discontinuation: Disease progression and declining performance status (N = 4), patient completed surgical removal of single site of disease and no additional chemotherapy was required due to absence of active disease elsewhere (N = 1), patient completed 6 cycles of planned palliative chemotherapy (N = 1), patient opted to pursue high intensity frequency ultrasound treatment but passed away prior to commencing local treatment (N = 1).

Supplementary Table S2C: Systemic treatment details for patient on 3rd line chemotherapy (N = 9).

	**Total number of subjects**	
	**N**	**%**
**Number of chemotherapy cycles completed**		
Median	3	
Range	1 – 8	
**Duration of total chemotherapy received (months)***		
Median	2.45	
Range	0.00 – 3.94	
**Nature of chemotherapy continuity**	**9**	**100**
Non-continuous treatment	6	67
Unknown	3	33
**Number of chemotherapy interruptions**		
Median	2	
Range	1 – 2	
**Total duration of chemotherapy interruptions (days)**		
Median	10	
Range	7 – 12	
**Common reasons for treatment interruptions**		
Covid infection	2	67
Patient choice	1	33
**Patterns of change in current chemotherapy or treatment continuation**	**9**	**100**
Discontinued all systemic therapy due to disease progression	1	11
Discontinued all systemic therapy due to disease progression and declining performance status	1	11
Switched to next line chemotherapy due to disease progression	4	44
Ongoing current chemotherapy as of last follow-up visit	3	33

* Refers to actual chemotherapy duration while excluding total treatment interruptions.

Supplementary Table S3A: Best overall response amongst unresectable patients following 1st line treatment with chemotherapy stratified by stage at initial diagnosis (N = 110).

	Total number of subjects	
	**N**	**%**
**Response status for borderline resectable PDAC patients**	**14**	**100**
Complete response[a]	4	29
Partial response[b]	2	14
Stable disease[c]	3	21
Progressive disease	4	29
Unevaluable	1	7
**Response status for locally advanced PDAC patients**	**22**	**100**
Complete response	1	5
Partial response[d]	8	36
Stable disease[e]	11	50
Progressive disease	1	5
Unevaluable	1	5
**Response status for localized disease followed by distant metastases patients**	**11**	**100**
Complete response[f]	2	18
Partial response[g]	2	18
Stable disease[h]	3	27
Progressive disease[i]	4	36
**Response status for de novo metastatic patients**	**63**	**100**
Complete response	4	6
Partial response[j]	28	44
Mixed response[k]	4	6

Stable disease	12	19
Progressive disease	11	17
Unevaluable	4	6

Abbreviations: PDAC, Pancreatic Ductal Adenocarcinoma.
[a]Patient underwent Whipple's procedure/total pancreatectomy after neoadjuvant chemotherapy (N = 3).
[b]Patient underwent Whipple's procedure after neoadjuvant chemotherapy (N = 1).
[c]Patient received radiotherapy to pancreatic primary followed by Whipple's procedure after neoadjuvant chemotherapy (N = 2).
[d]Patient underwent Whipple's procedure after neoadjuvant chemotherapy (N = 2). Patient received radiotherapy to pancreas after commencing chemotherapy (N = 2). Patient received radiotherapy to pancreas after commencing chemotherapy followed by surgical resection of the primary tumour (N = 1).
[e]Patient received radiotherapy to pancreas after commencing chemotherapy (N = 6).
[f]Patient underwent Whipple's procedure/surgical resection after commencing chemotherapy (N = 2).
[g]Patient underwent pancreatectomy/surgical resection after commencing chemotherapy (N = 2).
[h]Patient underwent Whipple's procedure after neoadjuvant chemotherapy (N = 1). Patient received radiotherapy to pancreas after commencing chemotherapy followed by surgical resection of the primary tumour (N = 1).
[i]Patient received radiotherapy to pancreas after commencing chemotherapy (N = 1).
[j]Patient received radiotherapy to right humerus after commencing chemotherapy (N = 1). Patient received radiotherapy to liver metastases and Whipple's procedure after completing long term chemotherapy (N = 1). Patient underwent surgical removal of metastatic lung lesion after commencing chemotherapy (N = 1). Patient received surgical resection of pancreatic primary tumour (N = 1). Patient received radiotherapy to the pancreatic primary tumour (N = 1).
[k]Patient received radiotherapy to brain metastases after commencing chemotherapy (N = 1).

Supplementary Table S3B: Best overall response amongst unresectable patients following 2nd line treatment with chemotherapy stratified by stage at initial diagnosis (N = 33).

	**Total number of subjects**	
	**N**	**%**
**Response status for borderline resectable PDAC patients**	**2**	**100**
Progressive disease	1	50
Unevaluable	1	50
**Response status for locally advanced PDAC patients**	**3**	**100**
Partial response	2	67
Stable disease	1	33
**Response status for localized disease followed by distant metastases patients**	**10**	**100**
Stable disease	3	30
Progressive disease	3	30
Unevaluable[a]	4	40
**Response status for de novo metastatic patients**	**18**	**100**
Complete response	1	6
Partial response	3	17
Stable disease	3	17
Progressive disease[b]	10	56
Unevaluable[c]	1	6

Abbreviations: PDAC, Pancreatic Ductal Adenocarcinoma.

[a]Patient received radiotherapy to liver metastasis after commencing chemotherapy (N = 1).

[b]Patient received radiotherapy to pancreas after commencing chemotherapy (N = 1).

[c]Patient received radiofrequency ablation to solitary liver lesion after commencing chemotherapy (N = 1).

Supplementary Table S3C: Best overall response amongst unresectable patient following 3rd line treatment with chemotherapy stratified by stage at initial diagnosis (N = 7).

	Total number of subjects	
	**N**	**%**
**Response status for localized disease followed by distant metastases patients**	**2**	**100**
Progressive disease	1	50
Unevaluable[a]	1	50
**Response status for de novo metastatic patient**	**5**	**100**
Stable disease	2	40
Progressive disease	3	60

[a]Patient received radiotherapy to liver metastases after commencing chemotherapy (N = 1).
